# Effectiveness of an Eye-Cervical Re-Education Program in Chronic Neck Pain: A Randomized Clinical Trial

**DOI:** 10.1155/2020/2760413

**Published:** 2020-02-26

**Authors:** Verónica Pérez-Cabezas, Carmen Ruiz-Molinero, Jose Jesús Jimenez-Rejano, Gema Chamorro-Moriana, Gloria Gonzalez-Medina, Raquel Chillon-Martinez

**Affiliations:** ^1^Department of Nursing and Physiotherapy, University of Cadiz, Cadiz 11009, Spain; ^2^Department of Physiotherapy, University of Seville, Seville 41009, Spain

## Abstract

**Objectives:**

Proprioceptive training is popularly applied as a therapeutic exercise method in physiotherapy. Its effects on pain and range of motion are only poorly evaluated. Therefore, this study assesses the effectiveness of proprioceptive training with an Eye-Cervical Re-education Program to decrease pain and increase the joint range in chronic neck pain patients. *Material and Methods*.

**Design:**

A randomized, no-blinded, controlled clinical trial. *Setting*. Physiotherapy consultation. *Participants*. 44 people were divided into two groups. *Interventions*. All patients were treated with a multimodal physiotherapy intervention. The experimental group was supplemented with an exercise program that included eye-cervical proprioception. *Outcomes*. The primary outcomes included pain pressure thresholds (upper trapezius, levator scapulae, and splenius capitis) and cervical range of motion. The secondary outcomes included pain measured by the Visual Analogical Scale and the McGillSpv Questionnaire.

**Results:**

The proprioception treatment was effective in reducing the pain pressure threshold in the right upper trapezius (*p*=0.001), left upper trapezius (*p*=0.001), left upper trapezius (*p*=0.001), left upper trapezius (*p*=0.001), left upper trapezius (*p*=0.001), left upper trapezius (

**Conclusions:**

The Eye-Cervical Re-education Program is effective at relieving pain pressure thresholds in the upper trapezius, right levator scapula, and left splenius capitis and especially effective for increasing the cervical range of motion. This trial is registered with NCT03197285 (retrospective registration).

## 1. Introduction

This study assesses chronic neck pain (CNP), a common problem in modern and industrialized countries [1] and among employed individuals [2]. Approximately 20% of the European adult population has chronic neck pain [1]. The estimated expenditure in pharmacology and diagnostic tests has significantly increased recently [3]; moreover, in addition to the physical and emotional burden of CNP, the financial cost to society is substantial, currently estimated at more than €200 billion per annum in Europe and $150 billion in the USA [1].

Adults with neck pain commonly experience hyperalgesia of cervical muscles, as evidenced by a reduced pressure pain threshold (PPT) [4]. Pain symptoms are thought to worsen in response to prolonged static muscle activity and/or repetitive job tasks, causing muscle metabolic disturbances [5]. The reduced range of neck motion (ROM) is another objective finding widely investigated in CNP [6, 7]. It could be argued that the optimal functioning of the cervical musculature is related to the ROM; changes in neck muscle activation that result in an altered stiffness distribution may affect cervical passive stability as well as the passive and active ROM [8].

Proprioceptive function accuracy has recently gained considerable attention in the description and assessment of CNP [9, 10]. The cervical spine has an important role in providing the proprioceptive input and this is reflected in the abundance of cervical mechanoreceptors and their central and reflex connections to the vestibular, visual, and central nervous systems [11]. Some investigators assume that proprioception deficit might be a factor predisposing to pain and injury via poor motor control [12].

Considering these findings, the goal of the present study focuses on proprioceptive training at the cervical level, a therapeutic option that targets maladaptive changes in patients suffering from neck pain; however, its effects on pain and function have been poorly evaluated [13].

The Eye-Cervical Re-education Program (ECRP) is a specific proprioceptive training program that includes head relocation exercise practice, gaze stability, eye follow, and eye/head coordination exercises [14]. This program has been considered in several studies [14–17]. Humphreys and Irgens compared eye-neck coordination exercises with nonintervention in the control group [15]. In Revel et al.'s study, the control intervention included the administration of symptomatic analgesics [14]. In other studies, the control intervention was another type of proprioception training [16, 17]. There was no homogeneity in the results obtained. In the studies where the control group had no exercise intervention, positive results were obtained in the repositioning of the head [14, 15]. Jull et al. compared two exercise regimes, one of them a proprioceptive exercise program, and the results for the joint position error improved in both groups but they were not in favour for any of them [16]. The findings of the authors who compared two different proprioceptive training programs were similar for the intensity of pain; both intervention groups demonstrated a significant reduction in average intensity but there was no difference between groups [16, 17].

To the best of our knowledge, the effect of the ECRP versus that of the multimodal physiotherapy intervention (MPI) efficacy has not been analyzed. Thus, this clinical trial aims to verify the effectiveness of the ECRP against an isolated multimodal physiotherapy intervention in increasing the pressure pain threshold and range of motion in the cervical area.

## 2. Materials and Methods

### 2.1. Study Design

This study was a no-blinded, randomized controlled clinical trial, with parallel groups and a blinded assessor. It was guided according to the CONSORT Statement guidelines. This research received approval from the Ethics Committee of the University of Seville (ref. 30062010). The authors confirm that all research was performed in accordance with the relevant guidelines of the Ethics Committee of the University of Seville. Participation in the study was voluntary. Patients were informed orally and in writing regarding the procedure to be conducted, and informed consent was obtained from all participants. The study was registered at clinicaltrials.gov (NCT03197285) (retrospective registration).

### 2.2. Participants

All participants were recruited in a private physiotherapy consultation in Cadiz (Spain) by a specialized physician in traumatology with more than 12 years of experience. The inclusion criteria were as follows: patients of both sexes, age between 20 and 50 years, with neck pain diagnosed by their physician greater than 3 months in duration and active or latent myofascial trigger points (MTrPs) in at least one of the following muscles: upper trapezius, levator scapulae, or splenius capitis. Both active and latent MTrPs were considered because latent MTrPs have been associated with the development of sensory-motor dysfunction and may contribute to different chronic musculoskeletal pain disorders [18]. Regarding exclusion, the authors considered dizziness syndrome, microwave contraindications, and analgesic currents (therapeutic procedures used), or posttraumatic, rheumatologic, neurological, infectious, or tumor cervical pain.

### 2.3. Study Settings

Two groups of subjects were established; the control group (CG) received multimodal physiotherapy intervention (MPI) as subsequently described, and the experimental group (EG) was treated with the MPI and an Eye-Cervical Re-education Program (ECRP) developed by Revel et al. [14]. All procedures were performed under similar environmental conditions, as they occurred in the consultation room reserved for this study (temperature, lighting, and morning time). The distribution within the groups was randomized using sealed envelopes.

### 2.4. Performance Protocol

A trained and blinded physiotherapist performed the initial and final assessments. This professional had more than 10 years of clinical experience. In the first session, self-reported outcomes were collected via the McGillQSpV and the Visual Analogue Scale (VAS). The following clinical tests were subsequently performed: range of motion (ROM) and pressure pain threshold (PPT). These clinical tests will be described in detail and show acceptable reliability, construct and discriminative validity.

The treatment was performed by a different physiotherapist than the evaluator. In this case, she could not be blinded because she had to apply the ECRP. The subjects received ten sessions on alternate days. All patients were treated with an MPI that consisted of thermotherapy (70 W continuous microwave 10 minutes), therapeutic massage (surface rubbing 5 minutes, 10 minutes of compression, and kneading massage and 2 minutes of final surface friction), and the application of analgesic currents (transcutaneous electrical nerve stimulation (TENS) using 4 × 4 cm self-adhesive silicone electrodes, symmetrical biphasic rectangular current, a 200 *µ*s width pulse, and a frequency of 1 Hz for 10 minutes. The patient should notice a slight vibration, without it being painful).

The ECRP developed by Revel et al. [14] had been also applied to patients in the experimental group.

#### 2.4.1. Eye-Cervical Re-education Program (ECRP)

This program includes 10 exercises that have proprioceptive reprogramming in the cervical area with the following phases:

- To stimulate ocular mobility without including the cervical movement: the patient was placed in the supine position, with the physiotherapist seated at the height of the head.
- *Exercise 1*. Activation of ocular muscles. Without moving the head, analytical exercises on the maximum amplitude of ocular movement towards the right, left, front, and feet were actively performed. The exercise was repeated 3 times, first with the eyes open and then with closed eyes.
- *Exercise 2*. The physiotherapist performed a passive mobilization of the cervical spine in rotation and flexo-extension, while the patient maintained the eyes at a fixed point located in the vertical direction. After memorizing this point, the exercise was repeated with eyes closed.
- To exercise cervical mobility with restricted eye movement. the patient is placed on a rotating stool. The ocular mobility is excluded with opaque glasses that exclusively allowed the foveal vision.
- *Exercise 3*. Analytical exercises on cervical mobility were actively performed. The goal was to keep the gaze on a target as far as possible in each of the directions. Each movement was repeated 3 times.
- *Exercise 4*. Global exercise of cervical movement. The patient follows with his eyes a complex geometric path or a graphic painted on the wall.
- *Exercise 5*. Cervical mobility work with the trunk. The patient fixed his gaze on a target on the wall, while the physiotherapist destabilized the trunk in all directions in a combined manner.
- *Exercise 6*. Head reposition exercise (first degree). The patient was placed in front of a mirror in the correct position. After memorizing this position, he had to make movements with his eyes closed (flexo-extension, rotations, and lateral-flexions). Without opening his eyes, the patient should try to return to the starting position. This exercise is repeated 10 times.
- *Exercise 7*. Head reposition exercise (second degree). This exercise is the same as the previous exercise except the physiotherapist destabilized the patient.
- Finally, we stimulate eye and neck movement coordination. The patient continued to sit on the stool, in this case, without the glasses.
- *Exercise 8*. Free coordination exercise. The physiotherapist stood in front of the patient with an object in his hand. The patient fixed his eyes on this object, which was directed by the physiotherapist with the intention of reaching the maximum amplitude in each of the movements. The duration of this exercise was one minute, and it was repeated twice. The amplitude of the movements performed by the physiotherapist depended on the condition of the patient but was applied throughout the joint range available.
- *Exercise 9*. Manual resistance coordination exercise. The physiotherapist stood behind the patient. The subject had to move in a requested direction and, in turn, the physiotherapist offered manual resistance to the movement. As in exercise 8, the amplitude and resistance depended on the physical condition of the patient. The duration was 2 minutes.
- *Exercise 10*. Oculocervical coordination and multidirectional manual stimuli work. Exercise 9 was repeated; however, instead of offering manual resistance, the physiotherapist performed soft imbalances on the patient's head. The duration was two minutes.

At the end of the last session, the same blinded evaluator performed the measurements in all subjects. Registered computerized medical records were used to collect demographic and clinical information on the patients.

### 2.5. Primary Outcome Variables

The primary outcome measures reported in this study included the cervical pressure pain threshold and cervical range of motion.

Pressure pain threshold (PPT) is a reliable outcome measure to measure pain [18]. The PPT was examined bilaterally at three sites, including over the upper trapezius, levator scapulae and splenius capitis (splenius capitis) muscles, using a digital algometer (JTech Medical Industries, ZEVEX Company) with a surface area of 1 cm^2^ at the round tip. To ensure the repeatability of the location for the subsequent assessment sessions, the PPT measurements were performed at the splenius capitis sites near the upper insertion of the trapezius muscle 2 cm lateral to the spinous process of the axis and on the levator scapulae muscle 2 cm above the lower insertion located in the upper medial border of the scapulae while patients were lying prone. Finally, measurements were obtained on the upper border of the trapezius muscle half-away between the midline and lateral border of the acromion [18]. The patient was instructed to state immediately when the pressure sensation (kg/cm^2^) turned into a pain sensation, at which point compression was terminated. After a rest of approximately 30 seconds, the next measurement was obtained [18]. Measurement of the PPT by an algometer has an intrarater reliability of 0.6–0.97 and an interrater reliability that varies from 0.4 to 0.98 [19].

Range of motion (ROM) was examined using a bubble inclinometer (Baseline Bubble Inclinometer, Fabrication Enterprises Inc., USA) for flexion/extension and lateral flexion and neck rotation. This test has satisfactory psychometric properties and may be recommended for clinical use [6] with ICC measurements for the intra- and interexaminer reliability that ranged from 0.80 to 0.93 (“good to excellent”) [20]. The participants were instructed to actively perform flexion, extension, right side bending, left side bending, right rotation, and left rotation movements three times each to identify the mean of the measurements.

### 2.6. Secondary Outcome Variables

The neck pain intensity measured on VAS and McGillQSpV was considered as secondary outcomes.

VAS reproducibility has been recognized in individual subjects (ICC = 0.97) [21]. On the other hand, Lazaro et al. [22] validated the McGill Questionnaire in the Spanish version, which consists of 67 adjectives grouped in 17 subscales. These subscales are grouped into four dimensions: sensory, emotional, evaluative, and miscellaneous.

### 2.7. Sample Size Determination

The sample size was calculated using the free Gpower3.1.9.2 software for a clinical trial. The sample used for the calculation was obtained from a pilot study with similar characteristics to the present study, considering as outcome measure “pressure pain threshold in the right trapezius” with a difference between groups of 0.15 kg/cm^2^ and with a size of effect *d* of 0.80 [23] and representing a large effect size [24], an *α* = 0.05 and *β* = 0.20, and a ratio between groups N2/N1 = 1. The calculations indicated a sample size of 42 subjects, with 21 subjects per group. After the estimation and given the characteristics of our convenience sampling, we considered it appropriate to include 22 subjects per group (44 subjects in total), which indicated an increase in the power of the study up to 82% (*β* = 0.18).

### 2.8. Data Analysis

Data were analyzed with SPSS Version 25.00 for Windows (IBM, Armonk, NY, USA). Shapiro–Wilk test was used to verify the normality of the main variables. This statistic was calculated by first considering all subjects together, followed by considering each group separately. A description of our data was provided, which includes the means and standard deviations of normally distributed variables and the medians and interquartile ranges for nonnormally distributed variables. In the case of the qualitative gender variable, the absolute frequencies and percentages of each category were calculated.

The initial homogeneity of the two intervention groups was subsequently checked for the age and pretest variables of all dependent variables. Student's *t*-test was used for independent samples.

To determine the effectiveness of MPI the Student's *t*-test was used for related samples and the Wilcoxon signed-rank test for pretest and posttest values. When we considered two groups, Student's *t*-tests were used; for the variables that did not fit the normal, the Mann–Whitney *U* test was used. We complemented the significance test with effect size calculation using Cohen's *d* ($d=2t/\sqrt{df}$; being *d* = the standardized mean difference, *t* = the student's *t*, and *df* = degrees of freedom) [24]. The variables that did not fit the normal effect size were calculated following Grissom's criteria [25].

An intention to treat analysis was performed. Statistical tests were conducted considering a 95% confidence interval (CI) (*p* < 0.05).

## 3. Results

### 3.1. Participants

**Figure 1:**
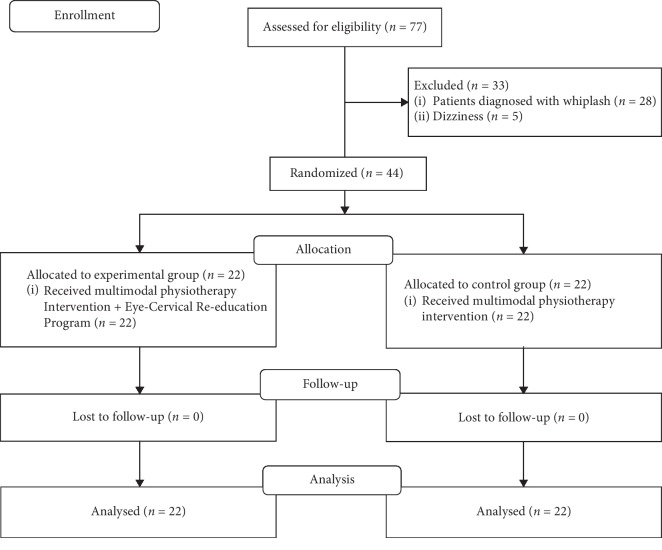
CONSORT flow diagram.

Seventy-seven subjects were initially considered; after applying the exclusion criteria, 44 were included (Figure 1). During the study, there was no loss of patients. The mean age was 39.68 years, with a standard deviation of 5.97, and the minimum and maximum values were 23 and 49 years, respectively. In both groups, 7 men (31.8%) and 15 women (68.2%) were included, and there was no gender difference between the groups. The two intervention groups did not show significant differences with respect to age and the pretest for all dependent variables (Table 1).

### 3.2. Primary Outcomes

The primary outcome measures reported in this study included cervical PPT and ROM. In the PPT, significant differences were identified between the groups in the right (*p*=0.001) and left (*p*=0.014) upper trapezius, right levator scapula (*p*=0.040), and the left splenius capitis (*p*=0.021), whereas for the left levator scapulae and the right splenius capitis, our results indicated no significant differences (*p* ≥ 0.05). For the ROM, statistically significant differences were observed in all ranges (*p* < 0.05). (Table 2).

### 3.3. Secondary Outcomes

There was no significant effect of ECRP on secondary outcomes compared with MPI.

In the case of the McGillQSpv, all items showed nonsignificant *p* values in a range between 0.05 and 0.98.

For the VAS, a *p*=0.07 was obtained with a mean difference of 6.32. These values cannot be considered significant (Table 3).

## 4. Discussion

The ECRP led to a significant increase in the pressure pain threshold over the right and left upper trapezius with a large effect size and over the left splenius capitis with a moderate effect size. The cervical range of motion was increased statistically significant in all movements measured with a large effect size.

To the best of our knowledge, this study is the first clinical trial to assess the effects of an ECRP compared to multimodal physiotherapy intervention for treating pressure pain sensitivity involving cervical MTrPs, and on the cervical range of motion in patients with chronic neck pain.

Previously, only three studies have investigated the effects of proprioceptive exercises on hyperalgesia of the superficial cervical musculature [17, 26, 27]. The results obtained in our study regarding the sensation of pain in the MTrPs do not coincide with the findings of Llunch et al. [26] and Bobos et al. [27] This discrepancy may be because proprioceptive exercises, which consist of craniocervical flexion training, did not have an eye-neck coordination component. Moreover, Izquierdo et al. [17] compared two proprioceptive programs: craniocervical flexion training versus proprioceptive training that includes oculomotor exercises. The proprioceptive training group showed an increase in the PPT over the right upper trapezius, right splenius, and right levator scapulae at postmonth 2 compared with the baseline. This finding reinforces that the best proprioceptive training option for the improvement of pain sensation in the MTrPs employs eye-neck coordination exercises. However, we must point out the nonblinding of our study due to the absence of a placebo exercise program. Therefore there could be a risk of bias in favour of the experimental group due to increased Physical Therapy time and increased number of manoeuvres per session.

Coordination between the deep and superficial flexors muscles is considered necessary for the safe progression of exercise in patients with neck pain [28]. It has been shown that the CNP population exhibits dysfunction of the deep cervical flexors [29]. Augmented activity of the superficial neck muscles may be compensatory for changed activation of the deep cervical muscles, [30] which, on the contrary, show signs of inhibition in neck pain [31]. Prolonged overactivity of the superficial cervical muscles may have deleterious effects on the properties of the muscle fiber membrane [32]. This may explain the improvements in the pressure pain sensitivity over trapezius MTrPs due to eye-neck coordination exercises. This might be due to an improved quality of cervical afferent input into the central nervous system afforded by eye-head coordination exercises that involve repeated, specific contractions of craniocervical musculatures, which contain high densities of muscle spindles. This training may improve muscle spindle function, translating to improved cervical proprioception [16]. This fact could be an important finding as some investigators assume that proprioception deficit might be a factor predisposing to pain and injury via poor motor control [12]. Thus, proprioceptive training incorporating eye-head coordination involves an improvement of proprioception that results in an increase of PPT over cervical superficial musculature.

Another question to consider could be the involvement of the cervical musculature in the stabilization of the gaze [33], noting the close relationship between the deep cervical extensors/rotators and horizontal eye movement [34]. Several studies have shown the relationship between the activity of the trapezius muscle and the functioning of the visual system. To maintain the stimulus target projected into the fovea, there is a need for compensatory eye movements and eye-neck (head) stabilization. A neural command should have an impact on the neck/shoulder muscle function via increased static muscle activity [35].

Considering the minimum clinically important difference (MCID) established in general for the PPT (a difference of at least 2.04 kg/cm^2^ between the means) [18], our results cannot be confirmed to be meaningful. However, it has not been identified in the MCID specific for the points considered in this study. Jorgensen et al. [6] determined the MCID for the PPT in the anterior tibialis (0.83 kg/cm^2^), left tibialis (0.9 kg/cm^2^), C5 right (0.07 kg/cm^2^), and C5 left (0.48 kg/cm^2^) in a chronic neck pain population but not in the trapezius, levator scapulae, and splenius capitis. These values are far from the values established for PPTs in general (2.04 kg/cm^2^). In future investigations, it is necessary to establish the MCID for the PPT typically measured in the chronic neck pain population. Then, we should return to the present analysis.

The cervical ROM was statistically significant in all movements measured with a large effect size and showed a substantially beneficial difference [6]. Deterich et al. [8] argue that cervical proprioceptive work involves better coordination of superficial and deep muscles, which leads to an increase in the ROM.

The values obtained in the VAS were not statistically significant and did not show a substantially beneficial difference. This instrument may be less sensitive with respect to measuring pain after cervical proprioception training alone [36]. This may be a result of the MPI applied in both groups. There are studies in which therapeutic massage [37] and TENS [38] obtained significant values in the VAS. These physiotherapy modalities were applied to all participants; therefore, the differences in the pre- and posttest values in each group were statistically significant, in contrast to the comparison between groups. This finding reinforces that the combination of exercise and manual therapy is the most efficacious of all conservative managements for CNP [30].

### 4.1. Limitations of the Study

After assessing the study, one limitation is the lack of follow-up in the medium and long term to assess the duration of the results obtained. Consequently, these data will be incorporated into future research. In addition, pathologies such as whiplash may be prospectively included because these patients present a greater number of alterations in the eye-cervical proprioception [39]. It would also be interesting to include other clinical tests related to cervical proprioception, such as joint position error or craniocervical flexion test [40].

The results of the study show some improvement in ROM (the difference in ROM improvement has been 2 to 5° in most directions, only in a few 7 to 10°, never more than 10). In addition, the study has failed to demonstrate improvement in neck pain and function. More research is needed, including variables to measure function such as the Neck Disability Index.

The method of sealed envelopes for randomization may add a risk of bias in favour of the experimental group. In future research methods that achieve better randomization should be used.

## 5. Conclusions

In conclusion, the ECRP is effective for increasing the pressure pain threshold in the right and left upper trapezius and left splenius capitis and especially effective for increasing joint cervical mobility. The ECRP implies a significant increase in the range of motion compared to the isolated application of a multimodal physiotherapy intervention.

## Figures and Tables

**Table 1:** Initial homogeneity of the two groups.

Variable	Control group median (IQR)	Experimental group median (IQR)	Sig.
Age (years)	39.86 (5.97)^*∗*^	39.50 (6.12)^*∗*^	*p*=0.843
PPT (kg/cm^2^)			
Right trapezius	2.77 (1.64; 3.50)	2.13 (1.38; 3.69)	*p*=0.431
Left trapezius	2.99 (1.85; 3.55)	1.93 (1.81; 3.48)	*p*=0.459
Right levator scapula	2.81 (1.89; 3.39)	2.47 (1.90; 3.36)	*p*=0.733
Left levator scapula	2.99 (2.07; 3.22)	2.59 (2.01; 3.45)	*p*=0.707
Right splenius capitis	1.97 (1.03; 1.91)	1.59 (1.02; 2.04)	*p*=0.972
Left splenius capitis	1.77 (0.61)^*∗*^	1.79 (0.75)^*∗*^	*p*=0.944
Flexion (°)	40.00 (28.75; 50.00)	35.00 (20.00; 60.00)	*p*=0.897
Extension (°)	20.00 (10.00; 46.25)	25.00 (10.00; 40.00)	*p*=0.933
Right side bending (°)	35.00 (25.00; 45.00)	30.00 (25.00; 40.00)	*p*=0.484
Left side bending (°)	40.00 (20.00; 45.00)	31.00 (25.00; 40.00)	*p*=0.226
Right rotation (°)	48.18 (23.02)^*∗*^	46.68 (16.09)^*∗*^	*p*=0.804
Left rotation (°)	49.23 (22.36)^*∗*^	46.09 (16.93)^*∗*^	*p*=0.633
McGill Questionnaire			
PRI-S	21.50 (19.00; 27.00)	23.50 (17.50; 27.00)	*p*=0.823
PRI-E	3.50 (1.75; 4.00)	4.00 (2.75; 4.25)	*p*=0.108
PRI-V	3,00 (3.00; 3.25)	3.00 (3.00; 4.00)	*p*=0.141
PRI-M	7.00 (7.00; 9.00)	7.00 (5.75; 9.00)	*p*=0.424
PRI-T	37.05 (7.69)^*∗*^	36.45 (7.77)^*∗*^	*p*=0.801
VAS	68.86 (15.67)^*∗*^	71.82 (16.51)^*∗*^	*p*=0.546

^*∗*^Mean and SD are shown. IQR: interquartile range (first and third quartiles). SD: standard deviation. PPT: pressure pain threshold. PRI-E: emotional pain rating index. PRI-M: miscellaneous pain rating index. PRI-S: sensory pain rating index. PRI-T: total pain rating index. PRI-V: evaluative pain rating index.

**Table 2:** Descriptive statistics of primary outcomes measurements for all participants and a comparison between the two measurements and the two groups.

Variable	Group	Pretest median (IQR)	Posttest median (IQR)	Within-group	Change score median (IQR)	Between-group	
				*p* value		*p* value	Effect size
PPT right trapezius	Control	2.77 (1.64; 3.50)	2.90 (1.89; 3.99)	*p*=0.002	−0.28 (0.33)^*∗*^	*p*=0.001	1.10^*∗∗*^
	Exp	2.13 (1.38; 3.69)	3.06 (1.86; 4.40)	*p* < 0.001	−0.59 (0.26)^*∗*^		
PPT left trapezius	Control	2.99 (1.85; 3.55)	3.20 (2.04; 3.81)	*p*=0.003	−0.30 (0.37)^*∗*^	*p*=0.014	0.78^*∗∗*^
	Exp	2.74 (1.65)^*∗*^	3.38 (1.16)^*∗*^	*p* < 0.001	−0.64 (0.49)^*∗*^		
PPT right levator scapula	Control	2.74 (1.02)^*∗*^	3.22 (1.12)^*∗*^	*p*=0.001	−0.48 (−0.56; −0.17)	*p*=0.040	0.36
	Exp	2.47 (1.90; 3.36)	3.08 (2.46; 4.79)	*p* < 0.001	−0.64 (−1.34; −0.34)		
PPT left levator scapula	Control	2.91 (1.08)^*∗*^	3.43 (1.15)^*∗*^	*p*=0.001	−0.39 (−0.78; −0.09)	*p*=0.549	0.11
	Exp	2.59 (2.01; 3.45)	3.15 (2.56; 5.08)	*p* < 0.001	−0.48 (−1.16; −0.05)		
PPT right splenius capitis	Control	1.67 (0.68)	2.06 (0.60)	*p* < 0.001	−0.45 (−0.51; −0.31)	*p*=0.916	0.02
	Exp	1.59 (1.02; 2.04)	1.91 (1.55; 2.63)	*p* < 0.001	−0.41 (−0.75; −0.25)		
PPT left splenius capitis	Control	1.78 (0.61)^*∗*^	2.10 (0.57)^*∗*^	*p* < 0.001	−0.33 (0.28)^*∗*^	*p*=0.021	0.72^*∗∗*^
	Exp	1.79 (0.75)^*∗*^	2.37 (0.74)^*∗*^	*p* < 0.001	−0.58 (0.41)^*∗*^		
Flexion (°)	Control	37.73 (15.94)^*∗*^	43.86 (14.55)^*∗*^	*p* < 0.001	5.00 (5.00; 6.25)	*p* < 0.001	0.69
	Exp	35.00 (20.00; 60.00)	62.50 (50.00; 70.00)	*p* < 0.001	20.00 (8.75; 31.25)		
Extension (°)	Control	20.00 (10.00; 46.25)	30.00 (18.75; 46.25)	*p*=0.002	2.50 (0.00; 5.00)	*p* < 0.001	0.76
	Exp	25.00 (10.00; 40.00)	45.00 (35.00; 50.00)	*p* < 0.001	15.00 (8.00; 25.00)		
Right side bending (°)	Control	35.00 (25.00; 45.00)	42.50 (30.00; 46.25)	*p* < 0.001	5.00 (3.75; 5.00)	*p* < 0.001	0.98
	Exp	30.00 (25.00; 40.00)	50.00 (45.00; 61.25	*p* < 0.001	20.00 (15.00; 25.00)		
Left side bending (°)	Control	40.00 (20.00; 45.00)	42.50 (30.00; 50.00)	*p*=0.001	7.50 (0.00; 15.00)	*p*=0.003	0.52
	Exp	32.05 (10.44)^*∗*^	54.32 (7.12)^*∗*^	*p* < 0.001	21.00 (13.75; 25.00)		
Right rotation (°)	Control	48.18 (23.02)^*∗*^	55.68 (22.11)^*∗*^	*p* < 0.001	5.00 (5.00; 10.00)	*p* < 0.001	0.92
	Exp	47.50 (30.00; 60.00)	85.00 (75.00; 86.25)	*p* < 0.001	31.50 (25.00; 45.00)		
Left rotation (°)	Control	49.23 (22.36)^*∗*^	59.55 (20.75)^*∗*^	*p* < 0.001	15.00 (5.00; 26.25)	*p*=0.019	0.41
	Exp	46.00 (30.00; 56.25)	85.00 (70.00; 90.00)	*p* < 0.001	35.00 (23.75; 43.50)		

^*∗*^Mean and SD are shown. ^*∗∗*^Cohen's *d*.

**Table 3:** Descriptive statistics of secondary outcomes measurements for all participants and a comparison between the two measurements and the two groups.

Variable	Group	Pretest median (IQR)	Posttest median (IQR)	Within-group	Change score median (IQR)	Between-group	
				*p* value		*p* value	Effect size
McGill Questionnaire							
PRI-S	Control	21.50 (19.00; 27.00)	4.00 (3.00; 5.25)	*p* < 0.001	19.14 (4.79)^*∗*^	*p*=0.881	0.05^*∗∗*^
	Exp	23.50 (17.50; 27.00)	3.00 (2.00; 4.00)	*p* < 0.001	18.91 (5.25)^*∗*^		
PRI-E	Control	3.50 (1.75; 4.00)	1.00 (1.00; 1.00)	*p* < 0.001	2.50 (1.00; 3.00)	*p*=0.051	0.33
	Exp	4.00 (2.75; 4.25)	1.00 (0.00; 1.00)	*p* < 0.001	3.00 (2.75; 4.00)		
PRI-V	Control	3,00 (3.00; 3.25)	1.00 (1.00; 2.00)	*p* < 0.001	2.00 (2.00; 2.00)	*p*=0.254	0.16
	Exp	3.00 (3.00; 4.00)	1.00 (1.00; 2.00)	*p* < 0.001	2.00 (2.00; 2.25)		
PRI-M	Control	7.00 (7.00; 9.00)	1.00 (1.00; 2.00)	*p* < 0.001	6.00 (6.00; 8.00)	*p*=0.981	0.004
	Exp	7.00 (5.75; 9.00)	0.00 (0.00; 1.00)	*p* < 0.001	5.00 (7.00; 8.00)		
PRI-T	Control	37.00 (31.75; 42.25)	8.00 (6.00; 9.00	*p* < 0.001	29.82 (7.33)^*∗*^	*p*=0.675	0.13^*∗∗*^
	Exp	36.45 (7.77)^*∗*^	5.73 (2.47)^*∗*^	*p* < 0.001	30.73 (6.95)^*∗*^		
VAS	Control	68.86 (15.67)^*∗*^	21.95 (11.19)^*∗*^	*p* < 0.001	46.91 (10.13)^*∗*^	*p*=0.075	0.55^*∗∗*^
	Exp	71.82 (16.51)^*∗*^	18.59 (10.42)^*∗*^	*p* < 0.001	53.23 (12.65)^*∗*^		

^*∗*^Mean and SD are shown. ^*∗∗*^Cohen's *d*. IQR: interquartile range (first and third quartiles). SD: standard deviation. PRI-E: emotional pain rating index. PRI-M: miscellaneous pain rating index. PRI-S: sensory pain rating index. PRI-T: total pain rating index. PRI-V: evaluative pain rating index.

## Data Availability

The data used to support the findings of this study are available from the corresponding author upon request.
